# Identification of Endogenous Control miRNAs for RT-qPCR in T-Cell Acute Lymphoblastic Leukemia

**DOI:** 10.3390/ijms19102858

**Published:** 2018-09-20

**Authors:** Monika Drobna, Bronisława Szarzyńska-Zawadzka, Patrycja Daca-Roszak, Maria Kosmalska, Roman Jaksik, Michał Witt, Małgorzata Dawidowska

**Affiliations:** 1 Institute of Human Genetics, Polish Academy of Sciences, 60-479 Poznań, Poland; monika.drobna@igcz.poznan.pl (M.D.); bronislawa.szarzynska-zawadzka@igcz.poznan.pl (B.S.-Z.); patrycja.daca-roszak@igcz.poznan.pl (P.D.-R.); maria.kosmalska@igcz.poznan.pl (M.K.); michal.witt@igcz.poznan.pl (M.W.); 2 Department, Silesian University of Technology, 44-100 Gliwice, Poland; roman.jaksik@polsl.pl

**Keywords:** miRNA (microRNA), T-cell acute lymphoblastic leukemia (T-ALL), normalization of miRNA expression in RT-qPCR, endogenous controls, reference genes, tissue analysis, cell lines

## Abstract

Optimal endogenous controls enable reliable normalization of microRNA (miRNA) expression in reverse-transcription quantitative PCR (RT-qPCR). This is particularly important when miRNAs are considered as candidate diagnostic or prognostic biomarkers. Universal endogenous controls are lacking, thus candidate normalizers must be evaluated individually for each experiment. Here we present a strategy that we applied to the identification of optimal control miRNAs for RT-qPCR profiling of miRNA expression in T-cell acute lymphoblastic leukemia (T-ALL) and in normal cells of T-lineage. First, using NormFinder for an iterative analysis of miRNA stability in our miRNA-seq data, we established the number of control miRNAs to be used in RT-qPCR. Then, we identified optimal control miRNAs by a comprehensive analysis of miRNA stability in miRNA-seq data and in RT-qPCR by analysis of RT-qPCR amplification efficiency and expression across a variety of T-lineage samples and T-ALL cell line culture conditions. We then showed the utility of the combination of three miRNAs as endogenous normalizers (hsa-miR-16-5p, hsa-miR-25-3p, and hsa-let-7a-5p). These miRNAs might serve as first-line candidate endogenous controls for RT-qPCR analysis of miRNAs in different types of T-lineage samples: T-ALL patient samples, T-ALL cell lines, normal immature thymocytes, and mature T-lymphocytes. The strategy we present is universal and can be transferred to other RT-qPCR experiments.

## 1. Introduction

### 1.1. Background

MicroRNAs (miRNAs) belong to the class of small noncoding RNAs, serving as negative regulators of gene expression at the posttranscriptional level [1,2]. They bind to 3′ untranslated regions (3′ UTRs) of their target mRNAs, leading to translational repression and thus gene silencing [3]. Aberrant expression of miRNAs contributes to diseases, including malignancies. In cancer, miRNAs may serve as proto-oncogenes, which target and silence tumor-suppressor genes (these miRNAs attain oncogenic properties when overexpressed), and as tumor-suppressor miRNAs, which negatively regulate the expression of oncogenes (the suppressive function of these miRNAs is lost or diminished due to their downregulation) [4]. Research on cancer-related miRNAs provides insight into the molecular pathogenesis of cancer and forms the basis for using miRNAs as diagnostic and prognostic markers and even as therapeutic targets. Thus, intracellular and extracellular (circulating exosomal) miRNAs are extensively studied in various cancer types [5,6,7], including hematologic malignancies [8,9,10].

The successful implementation of miRNAs as biomarkers in standardized diagnostic and treatment-stratification strategies depends on many factors, including the choice of a reliable method for miRNA profiling and an optimal strategy for normalization of miRNA expression [11,12].

### 1.2. Challenges of Normalization in RT-qPCR-Based miRNA Profiling

Despite a rapid increase in the use of high-throughput deep sequencing of the miRNA-transcriptome (miRNA-seq), RT-qPCR still remains the gold standard of miRNA profiling, used either as a primary method for expression analysis or for the validation of miRNA-seq results [13,14,15]. In contrast to miRNA-seq, which, due to a large number of features, allows for the use of distribution-based normalization techniques, RT-qPCR often requires the choice of appropriate endogenous controls to normalize miRNA expression levels. An optimal endogenous normalizer (EN) should be a gene or a combination of genes exhibiting stable and relatively abundant expression across all samples examined by RT-qPCR, regardless of their tissue of origin, the preanalytical procedures, or the time points analyzed [16].

The use of miRNAs (and not other types of RNAs) as ENs is currently the most commonly advocated strategy in RT-qPCR-based miRNA profiling [9,17,18,19,20]. The length of miRNA molecules excludes the possibility of using housekeeping gene transcripts, which are standard ENs for mRNA expression. Differences in length between miRNAs and mRNAs affect isolation yield, reverse transcription, and amplification efficiencies, while these should be similar for ENs and the studied transcripts [11]. Other small RNAs, such as small nuclear RNA (snRNA) and small nucleolar RNA (snoRNA), have frequently been used as endogenous controls in miRNA studies [8,9,21,22,23]. Yet, the length of these small RNAs is also different from miRNAs (60–200 nt for snoRNAs [24] and 150 nt for snRNAs [25], as compared to 20–24 for miRNAs [1]). miRNAs also differ structurally. Unlike other small RNA types, miRNAs contain 5′-phosphate and 3′-hydroxyl groups at their ends [1]. For these reasons, some commercially available technologies for RT-qPCR experiments, based on hydrolysis probes, implement solutions that hamper the use of ENs other than miRNAs. For example, the TaqMan Advanced miRNA technology provided by Thermo Fisher Scientific makes it possible to reverse-transcribe only the miRNA fraction during cDNA synthesis, excluding other small RNA types.

However, the question remains as to which miRNAs will serve as optimal ENs in a particular experiment. Universal endogenous control miRNAs are lacking, due to a large variation of miRNA expression profiles across various cell and tissue types [18]. This problem is particularly valid in research on miRNAs as potential cancer biomarkers. Therefore, instead of aiming to identify universal EN miRNAs, the focus should be on applying a universal methodology for the identification of optimal ENs for each particular study. Such a methodology should allow for a comprehensive evaluation of stability (using *in silico* and wet-lab approaches) of candidate ENs, selected and tested in a pilot phase, preceding the RT-qPCR expression analysis [12].

Here we present a strategy we applied for the identification of optimal EN miRNAs for miRNA profiling in T-cell acute lymphoblastic leukemia (T-ALL), an aggressive and highly heterogeneous type of hematologic cancer [26,27]. The study was driven by the shortage of data on comprehensive assessment of miRNAs as optimal ENs, specifically for cells of T-lineage.

### 1.3. miRNA Expression Profiling in T-Cell Acute Lymphoblastic Leukemia

In recent years, miRNAs have become an object of growing interest in the field of T-ALL research, due to their involvement in oncogenesis and their potential as candidate biomarkers [28,29]. Most of the data on miRNA expression in T-ALL patients and T-ALL cell lines comes from RT-qPCR-based studies [13,30,31], with only a few studies exploiting miRNA-seq technology [32,33].

One of the essential challenges in T-ALL research is the choice of a proper control material. The use of thymocytes separated from the thymus gland is widely approved [34,35,36] but technically demanding. Thus, other types of cells are often used as controls, including CD34+-enriched cells [37,38] or mature T-lymphocytes from peripheral blood or bone marrow [39,40]. Additionally, T-ALL cell lines serving as an *in vitro* model of this disease are often used for miRNA expression profiling. Importantly, miRNA expression in cell lines might be affected by culturing conditions, such as time of culture or medium composition [41,42]. Considering the diversity of cell and tissue types used in miRNA research in T-ALL, identifying stably expressed miRNAs to be used as optimal ENs for RT-qPCR is highly necessary.

The endogenous control miRNAs we analyzed can serve as the first-line choice for those dealing with miRNA expression in cells of T-lineage: T-ALL patient samples, T-ALL cell lines, normal thymocytes, and normal mature T-lymphocytes. The workflow we applied for the identification of optimal EN miRNAs was successfully used to validate our miRNA-seq results. Additionally, the strategy is universal and can be adapted to other RT-qPCR experiments for miRNA profiling in cancer samples and cell lines, and is potentially transferable to the identification of cancer biomarkers, cancer diagnostics, and therapy.

## 2. Results

**Figure 1:**
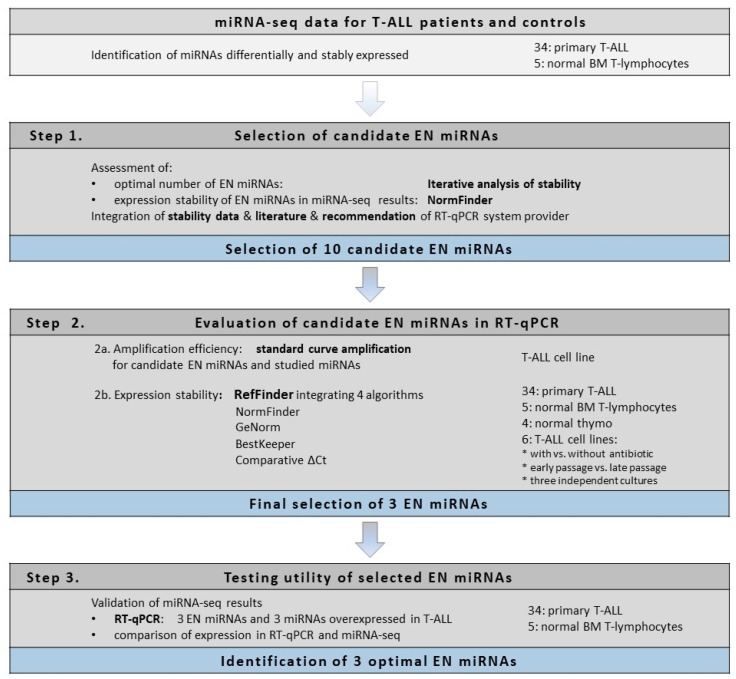
Workflow diagram illustrating strategy for identification of endogenous normalizer microRNAs (miRNAs) for RT-qPCR. BM, bone marrow; EN, endogenous normalizer; miRNA-seq, miRNA sequencing; T-ALL, T-cell acute lymphoblastic leukemia; thymo, thymocytes.

Here we present a strategy for the identification of miRNAs as optimal endogenous normalizers for RT-qPCR miRNA expression profiling used in T-lymphoid cells. The results of an miRNA-seq experiment were the starting point of our study, including 34 T-ALL samples and 5 samples of normal mature T-lymphocytes from bone marrow used as controls [43]. Based on miRNA-seq results, we identified a set of miRNAs that were differentially expressed between T-ALL samples and controls, including known and new potential oncogenic and tumor-suppressive miRNAs. We selected several of these miRNAs for validation by RT-qPCR, using TaqMan Advanced miRNA Assays (Thermo Fisher Scientific, Waltham, MA USA). To identify optimal EN miRNAs, we applied a stepwise strategy for the selection and evaluation of candidate ENs. The scheme of the workflow with respect to the type of analysis and material used in each step is shown in Figure 1.

### 2.1. Selection of Candidate Endogenous Normalizer miRNAs (Step 1)

In order to test how many reference miRNAs should be used, we created an iterative algorithm based on NormFinder (further referred to as iterative analysis of stability), which we used to select the best set of up to 100 miRNAs from our miRNA-seq data (Figure 1, Step 1). First, we conducted a standard NormFinder analysis and selected the most stable miRNAs out of a total of 1503 expressed in the analyzed cells. Then, we averaged the signals of the selected miRNAs with each of the remaining *N* - 1 miRNAs individually, each time repeating the NormFinder analysis. Based on that, we selected the best miRNA pair in terms of the stability index. This process was repeated by adding an additional miRNA from the remaining pool to the previously selected pair until we obtained a set of 100 unique miRNAs, which is already beyond any practical application in small-scale experiments such as RT-qPCR. The file including the code for this iterative algorithm, written in R programming language, is available in the Supplementary Materials (IterativeStability_v1.0.R).

**Figure 2:**
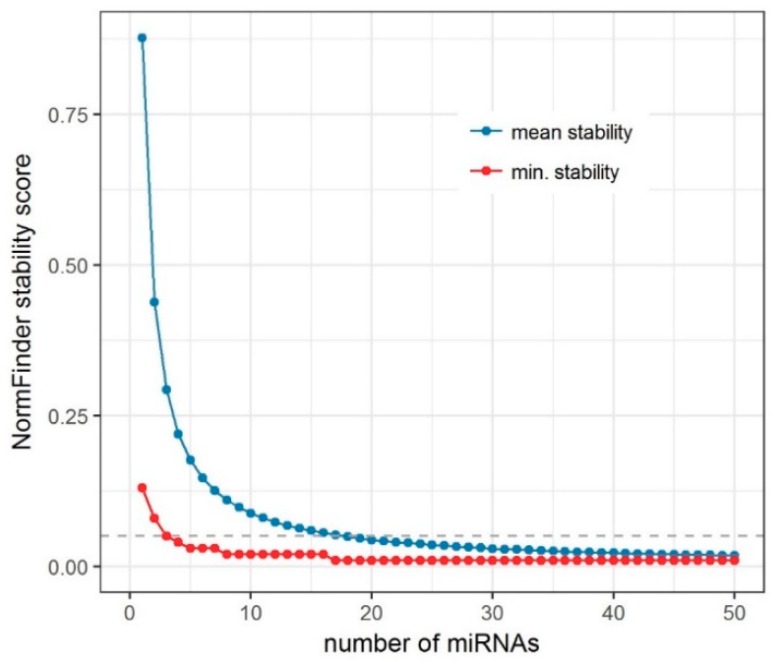
NormFinder stability scores relative to the number of miRNAs tested. For visualization purposes, we limited the plot to the results of the first 50 iterations. Beyond that point, no clear changes could be observed in the linear scale.

In Figure 2 we present the plots of the average NormFinder scores obtained for all sets in each iteration, and the minimum score that describes the best miRNA set of specific size. By adding additional miRNAs, we were able to reduce the score in general. However, the minimal score shows that the gain of incorporating one additional miRNA very quickly becomes negligible, being the highest for the first three iterations. Thus, in our dataset we show that the use of three miRNAs as ENs is reasonable, both for a fair representation of stably expressed miRNAs and for the cost of an RT-qPCR experiment.

In our experimental setting, the most optimal combination of three miRNAs identified by this iterative analysis of ENs was hsa-miR-1301-3p, hsa-miR-185-5p, and hsa-miR-30d-5p. Their stability scores according to NormFinder and mean read counts in miRNA-seq are shown in Supplementary Table S1. Due to relatively low read counts for two of these (hsa-miR-1301-3p and hsa-miR-185-5p), we decided to aim for three EN miRNAs in our RT-qPCR experiments, but searched for additional suitable candidates.

To accomplish this, we compared the list of the most stably expressed miRNAs in our miRNA-seq data with miRNAs recommended as suitable ENs for TaqMan Advanced miRNA Assays (www.thermofisher.com/advancedmirna). In addition, we searched through literature data regarding different tissues, including those of malignant origin [44,45,46,47,48]. Importantly, no such data regarding T-ALL and cells of T-lineage specifically are available so far. Thus, by integrating our miRNA-seq results with the recommendations for TaqMan assays and literature review, we selected 10 candidate EN miRNAs based on their stability scores and mean read counts in miRNA-seq, as illustrated in Figure 1, Step 1 and presented in Table 1. These 10 candidates were further comprehensively evaluated by RT-qPCR (Figure 1, Step 2).

### 2.2. Evaluation of Candidate Endogenous Normalizer miRNAs in RT-qPCR (Step 2)

Ten candidate EN miRNAs and three overexpressed miRNAs were tested by RT-qPCR for amplification efficiency of the assays and expression stability (Figure 1, Steps 2a and 2b, respectively). The slope and amplification efficiency data are shown in Table 2. Out of 13 assays, 11 exhibited amplification efficiency in the desired range of 90–110%, except for hsa-let-7f-5p and hsa-miR-181a-5p, with amplification efficiencies of 89% and 81%, respectively.

**Figure 3:**
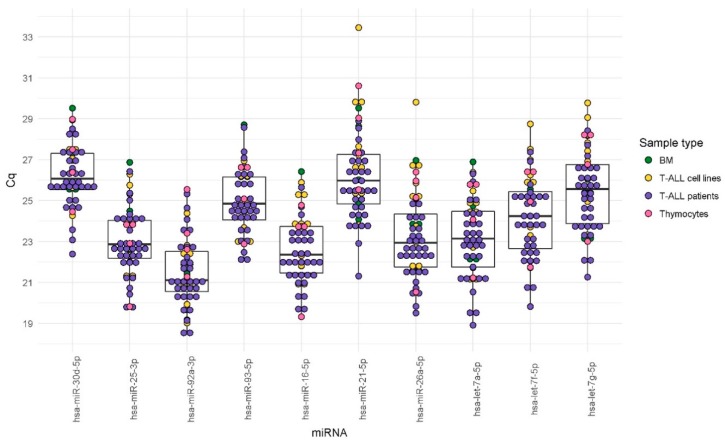
Overview of Cq values obtained by RT-qPCR for all samples with respect to the type of sample. BM, mature T-lymphocytes from normal bone marrow; thymocytes, normal precursors of T-cells. Dots represent mean raw Cq values for technical replicates of individual samples. Boxes correspond to the interquartile range (IQR) for each miRNA. Lines inside boxes indicate median Cq values. Candidate endogenous control miRNAs are ranked from left to right, according to increasing IQR value.

Mean raw Cq values and mean standard deviation (SD) across biological groups representing different types of material (T-ALL patient samples, T-ALL cell lines, normal mature T-lymphocytes of bone marrow, and normal thymocytes) are shown in Table 3. Mean raw Cq values and mean SD values obtained for T-ALL cell lines analyzed with the application of different culture types and conditions are shown in Supplementary Table S2. The variability of Cq values across all samples with respect to the type of material analyzed is presented for all candidate EN miRNAs in Figure 3.

**Figure 4:**
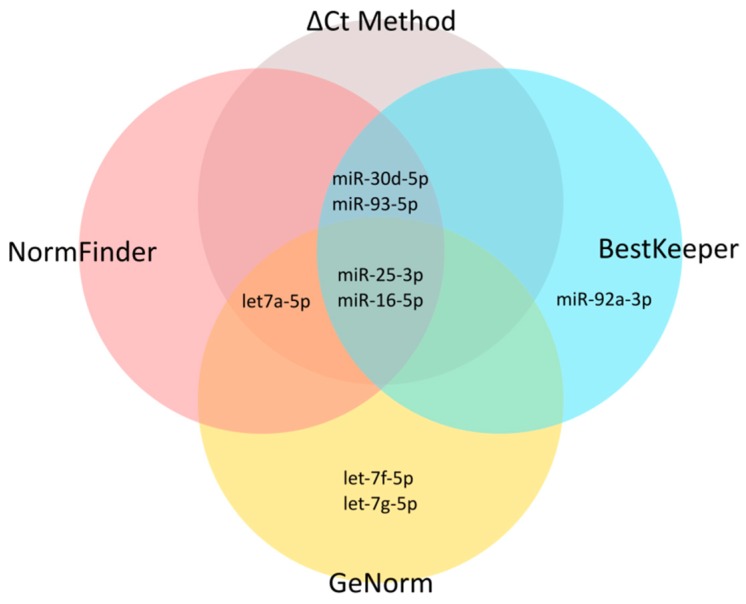
Venn diagram presenting the overlap between the five most stable candidate miRNAs indicated by each of four stability-testing algorithms.

Next, for these 10 candidate EN miRNAs, we analyzed expression stability by RT-qPCR across all samples using the RefFinder tool (http://leonxie.esy.es/RefFinder) (Figure 1, Step 2b) [49]. This online open source tool integrates four algorithms for expression stability assessment: NormFinder [50], geNorm [16], BestKeeper [51], and a comparative Delta C_T_ method [52]. Thus, RefFinder generates a comprehensive ranking of candidate ENs. The comprehensive stability ranking is shown in Table 4. The individual ranks generated by each algorithm separately are shown in Supplementary Tables S3–S6. The overlap between four stability-testing algorithms is presented in a Venn diagram in Figure 4. Out of the 10 candidate ENs, we finally selected three miRNAs: hsa-miR-16-5p, hsa-miR-25-3p, and hsa-let-7a-5p. The selection was based on the highest stability according to the RefFinder tool (Table 4), high overlapping between the four algorithms (Figure 4), and relatively high expression (low mean Cq values; equivalent to C_T_ values in Delta C_T_ method) across all samples (Table 3 and Figure 3).

### 2.3. Testing the Utility of Selected Candidate Endogenous Normalizer miRNAs (Step 3)

**Figure 5:**
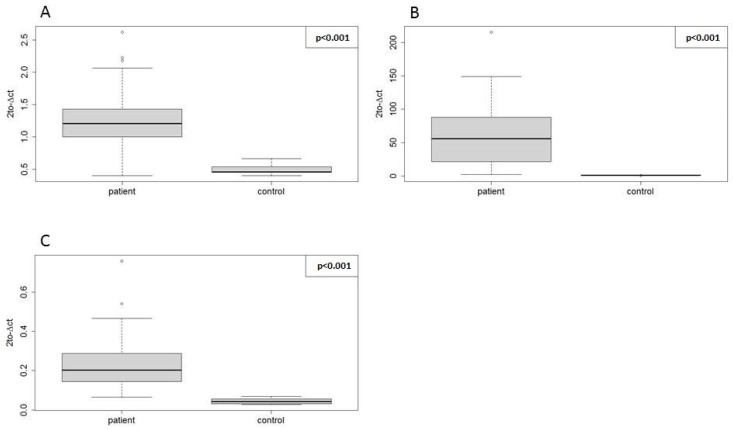
Normalized relative expression levels of miRNAs overexpressed in T-ALL vs. controls. (**A**) hsa-miR-20b-5p, (**B**) hsa-miR-181a-5p, and (**C**) hsa-miR-128-3p in patients (34 T-ALL samples) and in controls (5 samples of mature T-lymphocytes obtained from the bone marrow of healthy donors). Dots represent relative gene expression in individual samples. Upper and lower edges of boxes correspond to first (Q1) and third (Q3) quartiles, respectively. Lines inside boxes indicate median expression values. Whiskers extend to the smallest and largest observations within the 1.5-times interquartile range (IQR) from the box.

To test for the applicability of the three selected EN miRNAs, we used them in the RT-qPCR validation of our miRNA-seq results (Figure 1, Step 3). For the purposes of this study, we report the validation results for three miRNAs with an already-reported role in T-ALL biology: hsa-miR-128-3p, hsa-miR-181a-5p, and hsa-miR-20b-5p. We identified these miRNAs to be overexpressed in T-ALL (see Table 5), which is in line with their reported role as oncogenic miRNAs in this disease. In the RT-qPCR, we used the same samples that were used in miRNA-seq. Normalized relative expression levels of hsa-miR-20b-5p, hsa-miR-128-3p, and hsa-miR-181a-5p in patient samples and in normal controls are presented in Figure 5. For all three miRNAs tested by RT-qPCR, we observed statistically significant overexpression in T-ALL samples. The comparison of logarithmic fold change and *p*-values obtained by miRNA-seq and RT-qPCR is shown in Table 5.

To explain the possible reason for discrepancies between log2 fold changes of both methods, most striking in the case of hsa-miR-181a-5p, we tested for similarities between miRNA sequences. These may cause the nonspecific binding of primers and probes used in RT-qPCR. We performed a computational analysis of the global similarity between mature miRNA sequences, their seed sequences, and the Pearson’s correlation of expression based on our miRNA-seq results. The outcome of this analysis for hsa-miR-181a-5p is presented in Supplementary Table S7. The similarity of hsa-miR-181a-5p and its isoforms (isomiRs) was considerable, which may cause nonspecific annealing of primers and probes. It is noteworthy that the mean read counts of hsa-miR-181b-5p and hsa-miR-181d-5p in our miRNA-seq results were high enough (see Supplementary Table S7) to produce potential bias in the quantification of hsa-miR-181a-5p by RT-qPCR and explain the higher fold change value than that in miRNA-seq.

Regardless of the fold change discrepancies, the direction of expression changes and the statistical significance were consistent between both methods for all three miRNAs (see Table 5). Thus, the overexpression of hsa-miR-20b-5p, hsa-miR-128-3p, and hsa-miR-181a-5p in T-ALL patients relative to normal controls that we observed in the miRNA-seq experiment was successfully validated by RT-qPCR with the use of the selected EN miRNAs.

Taken together, the combination of criteria we adopted (i.e., comprehensively tested stability of expression and relative abundance of expression) allowed us to identify three miRNAs (hsa-miR-16-5p, hsa-miR-25-3p, and hsa-let-7a-5p) as optimal endogenous normalizers for RT-qPCR experiments in T-ALL samples.

## 3. Discussion

The choice of appropriate endogenous controls to normalize expression levels is one of the key factors greatly influencing the results of RT-qPCR expression profiling [53]. Optimal endogenous normalizers are crucial for the comparison of miRNA expression levels from different RT-qPCR experiments. Such interexperimental reproducibility of expression data is particularly important when miRNAs are considered as candidate biomarkers to be implemented in standardized diagnostic or treatment stratification procedures [54].

### 3.1. Strategy for the Identification of Optimal Endogenous Normalizer miRNAs for RT-qPCR

Here we present the strategy used for the identification of optimal endogenous normalizer (EN) miRNAs for RT-qPCR miRNA profiling in cells of T-lineage. The study design and experimental workflow are presented in Figure 1. The starting point of our study was the results of miRNA transcriptome profiling with use of next-generation sequencing (miRNA-seq) performed in pediatric T-ALL samples and normal controls [43]. First, using an iterative analysis of the stability of candidate ENs, we established three as an applicable number of miRNAs to be combined as ENs. Based on the expression stability in our miRNA-seq data, we selected 10 candidate EN miRNAs. Out of these, seven were recommended by Thermo Fisher Scientific as endogenous controls for TaqMan Advanced miRNA Assays (www.thermofisher.com/advancedmirna). Four miRNAs were also reported in the literature as suitable endogenous controls for different cancer samples [44,45,46,47,48]. To get insight into the potential utility of these candidate EN miRNAs in other experimental settings relative to cells of T-lineage, we included in our study four types of material commonly used in this research: primary T-ALL samples, normal mature T-lymphocytes from bone marrow, immature thymocyte samples, and six T-ALL cell lines. We also included varying cell line culture conditions for a more in-depth analysis of miRNA stability. Next, we analyzed the expression of these candidate EN miRNAs by RT-qPCR, and with the use of four algorithms we assessed the expression stability across all samples with respect to different material types. We selected three EN miRNAs (hsa-miR-16-5p, hsa-miR-25-3p, and hsa-let-7a-5p) exhibiting stable and abundant expression across all samples under study and successfully used them in the RT-qPCR validation of our miRNA-seq results.

Note that two of the three miRNAs we identified as the most optimal ENs for T-lineage cells, hsa-miR-25-3p and hsa-miR-16-5p, were among those proposed as universal ENs for TaqMan Advanced miRNA Assays (www.thermofisher.com/advancedmirna). These two miRNAs were also reported in the literature as optimal ENs used for miRNA expression profiling in cancer cells. Specifically, hsa-miR-25-3p was shown to be stably expressed in several human cancer cell lines, including cervical, breast, and colorectal cancer, acute lymphoblastic leukemia, and testicular embryonal carcinoma [45]. hsa-miR-16-5p was reported to be a suitable EN for miRNA expression profiling in malignant, benign, and normal breast tissues [44], and was also used as an EN for the profiling of exosomal miRNA expression in blood serum of patients with breast and gastric cancer [46,47,48]. The third miRNA we identified as an optimal EN, hsa-let-7a-5p, was reported to be suitable for miRNA expression analysis in human breast cancer [44]. Here we show that these three miRNAs are also optimal ENs for miRNA profiling in T-ALL.

Below, we discuss several important aspects of the selection of optimal normalizers for miRNA expression in light of the proposed strategy.

### 3.2. Number of miRNAs to Be Used as Endogenous Normalizers in RT-qPCR

There are several approaches to the normalization of expression data in RT-qPCR, such as the use of a single endogenous control gene, the geometric mean of expression of several endogenous controls, and the global mean of expression of all transcripts under study. Below, we discuss the issue of the number of endogenous normalizers to be used in RT-qPCR.

One of the approaches to normalization in RT-qPCR is the use of a single endogenous control gene. However, this approach may generate a large normalization error [16]. Therefore, normalization against a geometric mean of expression of several validated endogenous control genes is a more appropriate approach and greatly reduces the error rate [53,55].

Another possible strategy for RT-qPCR normalization is the use of a global mean expression value of all miRNAs under study (or genes, in the case of mRNA profiling) [56,57,58]. This approach was demonstrated by Mestdagh et al. to be accurate and reliable for miRNA profiling in RT-qPCR experiments [56]. In this study, the stability of a global mean was higher than the stability of commonly used endogenous control small RNAs. However, this approach is not suitable for every dataset, especially those in which a significant proportion of miRNAs show a positive correlation of signal intensities, which will limit the effectiveness of the approach. Additionally, a limitation of this method is the need to analyze a large and unbiased group of examined miRNAs or genes [56]. Another option for normalization of miRNA expression is a method based on the use of weight mean of miRNA expression to generate an artificial endogenous control used to calculate ΔCq values. The standard deviation of miRNA expression across all samples is used as a measure of stability, and the expression of each miRNA is weighted by its stability [59]. Yet, the utility of these approaches is limited to high- and medium-throughput expression profiling experiments like RT-qPCR-based arrays, and they are not suitable for small numbers of studied miRNAs or genes. Thus, the valid question is, what number of miRNAs should be used as ENs in a particular experiment?

To address this question, we created an algorithm for an iterative analysis of miRNA expression stability (Supplementary Material, IterativeStability_v1.0.R) and applied it to our miRNA-seq data. Several aspects should be considered when translating findings from miRNA-seq to an RT-qPCR experimental setting. The stability of miRNAs as measured by miRNA-seq depends not only on the intersample variability in miRNA concentrations, but also on the accuracy of the measurement method. The accuracy depends on the RNA isolation, the library preparation, the process of sequencing, and the data processing algorithms, including the data standardization strategy and its assumptions [17]. Furthermore, the expression of low-level miRNAs might be significantly affected by the sampling process, caused by the limited capacity of the flow-cell used in the sequencing process, as in RNA-seq [60]. For these reasons, even the most stable miRNAs will show significant variance between samples, caused by noise of technical origin. In order to reduce this effect, it is often beneficial to combine several endogenous reference RNAs for the normalization of expression data in RT-qPCR. The goal is to establish the number and optimal set of miRNAs to be combined. Using our miRNA-seq data, we selected the best *k*-number of miRNAs. However, in other experiments, a *k* + 1 number of combined miRNAs might provide a more stable signal. In general, in this iterative approach the stability is expected to increase as *k* increases, until we reach the point at which we start to incorporate differentially expressed miRNAs. However, in most cases, this will happen long after we exceed the practical limits of the number of miRNAs that can be used as reference in RT-qPCR experiments.

In our iterative analysis of miRNA expression stability, we demonstrated that the use of more than three EN miRNAs could provide only a limited increase of stability. The advantage of combining more miRNAs is likely disproportionate in view of additional cost. The use of three miRNAs seems to give a reasonable balance between good stability for endogenous normalization and cost-effectiveness of the study.

### 3.3. Expression Stability Relative to Sample Type and Culture Conditions

In our study, we analyzed the expression stability of 10 candidate EN miRNAs in several types of material commonly used in research on T-lineage cells, both normal and malignant. We included T-ALL samples, normal thymocytes, normal mature T-lymphocytes, and six T-ALL cell lines, cultured in varying conditions (as presented in Figure 1 and described in detail in the Materials section). We did not observe significant differences in the expression levels of candidate EN miRNAs between different types of biological material tested, as presented in Table 3. This observation also applies to T-ALL cell line samples cultured in different conditions, as presented in Supplementary Table S2. The variability of expression of these miRNAs is rather sample-dependent, as illustrated in Supplementary Figure S1.

We also demonstrated that our three selected EN miRNAs were suitable for miRNA profiling across different T-ALL cell lines and varying culture conditions by evaluating the relative expression of hsa-miR-128-3p in these samples in reference to normal bone marrow T-lymphocytes. As shown in Supplementary Figure S2, the hsa-miR-128-3p expression levels were very close in the MOLT-4 cells cultured with and without antibiotic. Similarly, the time of culture (early vs. late passage analyzed in the case of the CCRF-CEM cell line) did not affect the expression level of this miRNA. We observed the difference in fold change in one of the three independent cultures of Jurkat cells and, naturally, differences of fold change values across different cell lines. Nevertheless, using our three EN miRNAs, we showed that hsa-miR-128-3p was overexpressed in all six T-ALL cell lines, which is in line with literature data [35,61].

### 3.4. RT-qPCR Validation of miRNA-Seq Results

One of the discrepancies between miRNA-seq and RT-qPCR results when used for validation purposes is the difference of fold change values observed for differentially expressed miRNAs [62]. This can be explained by the fact that the operating principles for the two methods of quantification are different. miRNA-seq is based on the read count number of a particular transcript, whereas RT-qPCR is based on hybridization with primers and hydrolysis probe and amplification. The design of amplification primers and probes is particularly challenging in the case of miRNAs due to the fact that mature miRNA sequences are short. In combination with the high similarity between sequences of isomiRs, this limits the options for the optimal design of a miRNA assay to ensure the highest sensitivity and specificity [17]. These factors can potentially lead to nonspecific hybridization and eventually to biased RT-qPCR results as compared to miRNA-seq [62]. In our experimental setting, the difference in the fold change values was most striking in hsa-miR-181a-5p, as shown in Table 5. Based on the analysis of similarity between mature miRNA sequences, their seed sequences, and the Pearson’s correlation of expression in our miRNA-seq data, we concluded that this discrepancy might be attributed to high similarity of hsa-miR-181a-5p and its isomiRs, as presented in Supplementary Table S7.

Nevertheless, the direction of expression change and statistical significance were concordant between the two methods for all three miRNAs, which proves the positive validation of miRNA-seq findings with RT-qPCR using our EN miRNAs.

## 4. Materials and Methods

### 4.1. Materials

Bone marrow samples of 34 pediatric T-ALL patients and 5 age-related healthy donors were collected in Polish pediatric hemato-oncology centers. The study was approved by the Ethics Committee of the Medical University of Silesia (KNW/0022/KB1/153/I/16/17, 3 Octorber 2017). Informed consent was obtained in accordance with the Declaration of Helsinki. T-ALL cells and normal T-lymphocytes were selected from the samples by immunomagnetic separation. Four RNA samples obtained from CD34+ and CD4+CD8+ normal thymocyte subsets were a kind gift from Prof. Pieter van Vlierberghe (Center for Medical Genetics Ghent, Ghent University, Belgium). The samples were obtained and processed as described previously [32]. Six T-ALL cell lines—Jurkat, DND-41, CCRF-CEM, BE-13, P12-Ichikawa, and MOLT-4—were purchased from the Leibniz Institute DSMZ–German Collection of Microorganisms and Cell Cultures. To gain insight into miRNA stability under different culture conditions, we applied culture/harvesting settings as follows. All cell lines were harvested for RNA extraction at the fifth (early) passage. Additionally, CCRF-CEM cells were also harvested in the twentieth (late) passage. MOLT-4 cells were harvested after culture in antibiotic-free medium as well as after standard antibiotic-including culture, applied to all cell lines. The Jurkat cell line was harvested after 3 independent cultures in standard conditions. Details of T-ALL and bone marrow sample preparation, T-ALL cell line culture conditions, and RNA isolation are included in the Supplementary Materials and Methods.

### 4.2. miRNA-Seq

miRNA-seq was conducted by Exiqon (NGS Services Exiqon, Denmark) using NextSeq500 Illumina, with 10 million reads per sample, read length: 51 bp single-end; reference annotation: GRCh37. Raw sequencing reads were adapter-trimmed using Cutadapt (version 1.11) and aligned with Bowtie (version 1.2.2) to a modified version of miRBase (version 21) created according to the miRge specifications [63]. Differentially expressed miRNAs were selected using edgeR [64].

### 4.3. Reverse-Transcription and RT-qPCR Amplification Conditions

Total RNA, including miRNA fraction, was reverse-transcribed using an adapter-based TaqMan Advanced miRNA cDNA Synthesis Kit (Thermo Fisher Scientific, Waltham, MA, USA) with preamplification step according to the manufacturer’s protocol. Predesigned hydrolysis probe-based TaqMan Advanced miRNA Assays and TaqMan Fast Advanced Master Mix (Thermo Fisher Scientific) were used. The TaqMan assay IDs for the miRNAs are shown in Table 2. The reactions were performed using a 7900HT Fast Real-Time PCR System with 96-well block module (Applied Biosystems, currently part of Thermo Fisher Scientific, Waltham, MA, USA). All RT-qPCR reactions were performed in 3 technical replicates for each sample.

### 4.4. Amplification Efficiency

Standard curve analysis was applied to test the amplification efficiency of all assays (candidate EN miRNAs and miRNAs overexpressed in T-ALL). Standard curves were generated by 10-fold serial dilutions (10^0^–10^−5^) of cDNA, reverse-transcribed from RNA obtained from the DND-41 cell line.

### 4.5. Analysis of Gene Expression

For the relative quantification of gene expression, a comparative C_T_ method (ΔΔ C_T_) was used [65]. Statistical analysis was performed with Data Assist Software (v. 3.01; Thermo Fisher Scientific, Waltham, MA USA). The significance of differences in expression level between patients and controls was tested using a 2-tailed Student′s *t*-test, with *p* < 0.05 indicating statistical significance. Results are presented as boxplots. Whenever relevant, C_T_ values are further referred to as Cq values, in accordance with the Minimum Information for Publication of Quantitative Real-Time PCR Experiments guidelines [11]. Significance of Cq differences between biological groups representing different types of biological material used in the study were tested with one-way ANOVA and Benjamini and Hochberg correction to adjust for multiple testing (*p*-*adj*).

### 4.6. Analysis of Expression Stability

NormFinder software (https://moma.dk/normfinder-software) [50] was used to assess the stability of expression across all samples in miRNA-seq results. For stability testing by RT-qPCR, the Cq data collected for each candidate EN miRNA for each sample were used as input data for the RefFinder tool (http://leonxie.esy.es/RefFinder), integrating 4 algorithms for the assessment of expression stability: NormFinder [50], geNorm [16], BestKeeper [51], and a comparative Delta C_T_ method [52]. Stability ranks were generated comprehensively and individually from the 4 independent stability-assessing algorithms.

### 4.7. Analysis of the Similarity of Mature miRNA Sequences

Mature miRNA sequences were downloaded from miRBase v.21 and compared pairwise using the Needleman–Wunsch global alignment algorithm, as implemented in the pairwise Alignment function of the Biostrings R package (version 2.48). Seed sequences, defined as the nucleotides at positions 2–7 of the mature miRNA sequences, were compared separately, retaining only information about ideal matches of the compared sequences. Expression levels between individual miRNAs were compared pairwise using Pearson’s correlation coefficient.

## 5. Conclusions

Herein we present a strategy for the identification of miRNAs as optimal endogenous normalizers for the RT-qPCR profiling of miRNA expression in T-ALL cells and normal cells of T-lineage. Our strategy includes a computational approach to assess the applicable number of miRNAs to be used for the normalization of RT-qPCR results. The identification of optimal endogenous normalizers was based on a comprehensive analysis of miRNA expression stability in both miRNA-seq data and RT-qPCR, complemented by an analysis of RT-qPCR amplification efficiency and an analysis of expression across a variety of sample types and cell culture conditions. We showed the utility of the combination of three endogenous normalizers (hsa-miR-16-5p, hsa-miR-25-3p, and hsa-let-7a-5p) for successful validation of our miRNA-seq results.

The panel of 10 miRNAs, particularly the three miRNAs we identified as optimal endogenous normalizers, might serve as the first-line choice for those dealing with RT-qPCR expression analysis of miRNAs in different types of T-lineage samples: patient samples, T-ALL cell lines, normal thymocytes, and mature T-lymphocytes. So far, no data have been available on the comprehensive evaluation of candidate endogenous normalizer miRNAs regarding T-ALL and normal T-cells. Thus, our results fill this research gap, while the strategy we propose, including the algorithm we created for the assessment of expression stability, might be used more universally and transferred to other experimental settings concerning miRNA profiling with the use of RT-qPCR.

## Figures and Tables

**Table 1:** Candidate endogenous normalizer miRNAs.

Candidate EN miRNAs	Criteria for Selection			
miRNA Name	Stability in miRNA-Seq		Thermo Fisher Scientific Recommendation	Literature Data
	Stability Score *	Mean Read Count		
hsa-let-7a-5p	0.25	330,280	–	[44]
hsa-miR-30d-5p	0.25	98,732	–	–
hsa-miR-92a-3p	0.31	630,854	Suitable endogenous control for tissue samples	–
hsa-miR-93-5p	0.36	9838	Suitable endogenous control	[45]
hsa-let-7f-5p	0.37	326,028	–	–
hsa-miR-25-3p	0.45	108,466	Suitable endogenous control	[45]
hsa-miR-26a-5p	0.5	176,895	Suitable endogenous control for breast and heart tissue	–
hsa-miR-21-5p	0.5	116,490	Suitable endogenous control for tissue samples	–
hsa-miR-16-5p	0.52	5746	Suitable endogenous control	[46,47,48]
hsa-let-7g-5p	0.56	194,895	Suitable endogenous control	–

* Stability score according to NormFinder software. The stability score value is inversely proportional to the stability of gene expression.

**Table 2:** Amplification efficiency of miRNA assays.

miRNA Name	TaqMan Advanced miRNA Assay Name	Standard Curve		Amplification Efficiency (%)
		Slope	R2 (Correlation Coefficient)	
**Candidate EN miRNAs**				
hsa-let-7a-5p	478575_mir	−3.292	0.991	101
hsa-miR-30d-5p	478606_mir	−3.371	0.996	98
hsa-miR-92a-3p	477827_mir	−3.549	0.994	91
hsa-miR-93-5p	478210_mir	−3.371	0.996	98
hsa-let-7f-5p	478578_mir	−3.626	0.985	89
hsa-miR-25-3p	477994_mir	−3.533	0.994	92
hsa-miR-26a-5p	477995_mir	−3.558	0.997	91
hsa-miR-21-5p	477975_mir	−3.516	0.994	92
hsa-miR-16-5p	477860_mir	−3.582	0.997	90
hsa-let-7g-5p	478580_mir	−3.338	0.959	99
**Selected miRNAs Overexpressed in T-ALL vs. Control**				
hsa-miR-181a-5p	477857_mir	−3.872	0.966	81
hsa-miR-128-3p	477892_mir	−3.406	0.99	97
hsa-miR-20b-5p	477804_mir	−3.570	0.997	91

**Table 3:** Mean raw Cq and standard deviation (SD) values for candidate endogenous normalizer miRNA across analyzed samples with respect to biological groups.

miRNA	All Samples		T-ALL Samples		Normal BM T-Lymphocytes		Thymocytes		T-ALL Cell Lines		*p*-*adj*
	Cq	SD	Cq	SD	Cq	SD	Cq	SD	Cq	SD	
hsa-miR-92a-3p	21.54	1.68	21.38	1.61	20.37	0.92	23.22	1.78	21.65	1.53	0.173
hsa-miR-16-5p	22.58	1.66	22.18	1.42	23.61	1.79	22.89	2.42	23.40	1.82	0.192
hsa-miR-25-3p	23.00	1.64	22.70	1.51	24.11	1.76	22.60	1.90	23.67	1.83	0.240
hsa-let-7a-5p	23.08	1.91	22.66	1.83	23.98	1.99	24.22	2.15	23.73	1.89	0.240
hsa-miR-26a-5p	23.17	2.07	22.48	1.42	23.57	2.21	24.51	2.70	24.99	2.50	0.028
hsa-let-7f-5p	24.15	1.96	23.69	1.77	24.26	2.07	24.88	2.20	25.43	2.15	0.192
hsa-miR-93-5p	24.89	1.55	24.76	1.54	26.10	1.64	25.31	1.77	24.63	1.47	0.308
hsa-let-7g-5p	25.46	1.95	24.98	1.72	25.18	2.12	26.55	2.46	26.82	2.02	0.163
hsa-miR-21-5p	26.16	2.20	25.55	1.61	26.62	2.36	28.13	2.19	27.58	2.80	0.064
hsa-miR-30d-5p	26.18	1.49	25.91	1.38	26.98	1.62	26.83	1.90	26.59	1.56	0.308

Cq and SD values represent mean values across biological replicates. Significance of Cq differences between biological groups was tested with one-way ANOVA and Benjamini and Hochberg correction to adjust for multiple testing (*p*-*adj*). BM, bone marrow.

**Table 4:** RefFinder comprehensive ranking score of miRNA expression stability.

miRNA Name	Comprehensive Ranking Stability Score
hsa-miR-16-5p	2.11
hsa-miR-30d-5p	2.71
hsa-miR-25-3p	2.99
hsa-let-7g-5p	3.98
hsa-let-7a-5p	4.36
hsa-miR-93-5p	4.53
hsa-let-7f-5p	4.58
hsa-miR-92a-3p	5.66
hsa-miR-21-5p	8.74
hsa-miR-26a-5p	10

The stability score value is inversely proportional to the stability of gene expression.

**Table 5:** Validation of expression levels for selected miRNAs with oncogenic role in T-ALL.

miRNA Name	miRNA-Seq		RT-qPCR	
	Log2 Fold Change	*p*-Value	Log2 Fold Change	*p*-Value
hsa-miR-128-3p	2.814	<0.001	2.373	<0.001
hsa-miR-181a-5p	2.362	<0.001	5.951	<0.001
hsa-miR-20b-5p	3.522	<0.001	1.329	<0.001

## Supplementary Materials

Supplementary materials can be found at http://www.mdpi.com/1422-0067/19/10/2858/s1.

## Abbreviations

BM	bone marrow
Cq	quantification cycle ( nomenclature in accordance with Minimum Information for Publication of Quantitative Real-Time PCR Experiments, MIQE guidelines); Cq is equivalent to C_T_ (threshold cycle) which is only used, when referred to ΔΔ C_T_ method implemented in Data Assist Software version 3.01 (Thermo Fisher Scientific) or Comparative Delta C_T_ method; these methods has retained their original names
EN	endogenous normalizer
IQR	interquartile range
isomiR	miRNA isoform
miRNA-seq	next-generation sequencing of miRNA-transcriptome
Q1	first quartile
Q3	third quartile
SD	standard deviation
snoRNA	small nucleolar RNA
snRNA	small nuclear RNA
T-ALL	T-cell acute lymphoblastic leukemia
thymo	thymocytes
