# Supplementary material for: Identification of Endogenous Control miRNAs for RT-qPCR in T-Cell Acute Lymphoblastic Leukemia
Source: Int J Mol Sci. 2018 Sep 20;19(10):2858. doi: 10.3390/ijms19102858 (PMC6212946; doi:10.3390/ijms19102858)
Supplement: Supplementary file 1 [file ijms-19-02858-s001.zip › 2018-08-06 Drobna et al. _Suppl. Materials & Methods.docx]

Identification of endogenous control miRNAs
for RT-qPCR in T-cell acute lymphoblastic leukemia

Monika Drobna ^1^, Bronisława Szarzyńska-Zawadzka ^1^, Patrycja Daca-Roszak ^1^, Maria Kosmalska ^1^, Roman Jaksik ^2^, Michał Witt ^1^, Małgorzata Dawidowska ^1^*

SUPPLEMENTARY MATERIALS AND METHODS

Primary Pediatric T-ALL Samples and Normal Mature T-lymphocytes

Bone marrow samples of 34 pediatric T-ALL patients and 5 age-related healthy donors were collected in Polish pediatric hematology, oncology and stem cell transplantation centers. Mononuclear cells were isolated from bone marrow using density gradient centrifugation on Gradisol L (Aqua-Med). For T-ALL samples with blast content lower than 90% and for all bone marrow samples, the negative selection of T-ALL cells/T-lymphocytes by immunomagnetic separation was performed with Human T Lymphocyte Enrichment Set-DM (Becton Dickinson). The T-ALL/T-lymphocyte samples were stored in Lysis Solution (miRCURY RNA Isolation Kit, Exiqon) in -80°C until RNA isolation.

Thymocyte Subset Selection

Four RNA samples obtained from CD34+ and CD4+CD8+ normal thymocyte subsets were a kind gift from Prof. Pieter van Vlierberghe lab (Center for Medical Genetics Ghent, Ghent University, Belgium). The samples were obtained and processed as described previously (Wallaert et al. *Sci. Rep.* **2017**, *7*).

T-ALL Cell Lines

Six T-ALL cell lines: JURKAT, DND-41, CCRF-CEM, BE-13, P12-ICHIKAWA and MOLT-4 were purchased from the Leibniz Institute DSMZ-German Collection of Microorganisms and Cell Cultures, Germany. The cells were cultured in RPMI-1640 medium (Thermo Fisher Scientific) with 10% of fetal bovine serum (Thermo Fisher Scientific) and 1% of penicillin-streptomycin solution (Sigma Aldrich), in 37°C and in 5% CO2 atmosphere. All cell lines were harvested for RNA extraction at the fifth (early) passage. The JURKAT cell line was harvested after three independent cultures in standard conditions. Additionally, CCRF-CEM cells were also harvested in the twentieth (late) passage, and MOLT-4 cells were also harvested after culture in antibiotic-free medium. Three million cells from each culture was collected for RNA isolation.

RNA Extraction, Quantification and Quality Control

Total RNA from primary T-ALL, cell lines, and mature T-lymphocyte samples was extracted with the recovery of small RNA fraction, using miRCURY RNA Isolation Kit (Exiqon), according to the manufacturer’s protocol. RNA concentration was determined by fluorescent quantification with Qubit HS RNA Assay Kit (Thermo Fisher Scientific) using Quantus Fluorometer (Promega). The quality control of RNA was conducted with the use of 2100 Bioanalyzer (Agilent Genomics) and 4200 Tapestation (Agilent Genomics).

SUPPLEMENTARY FIGURES


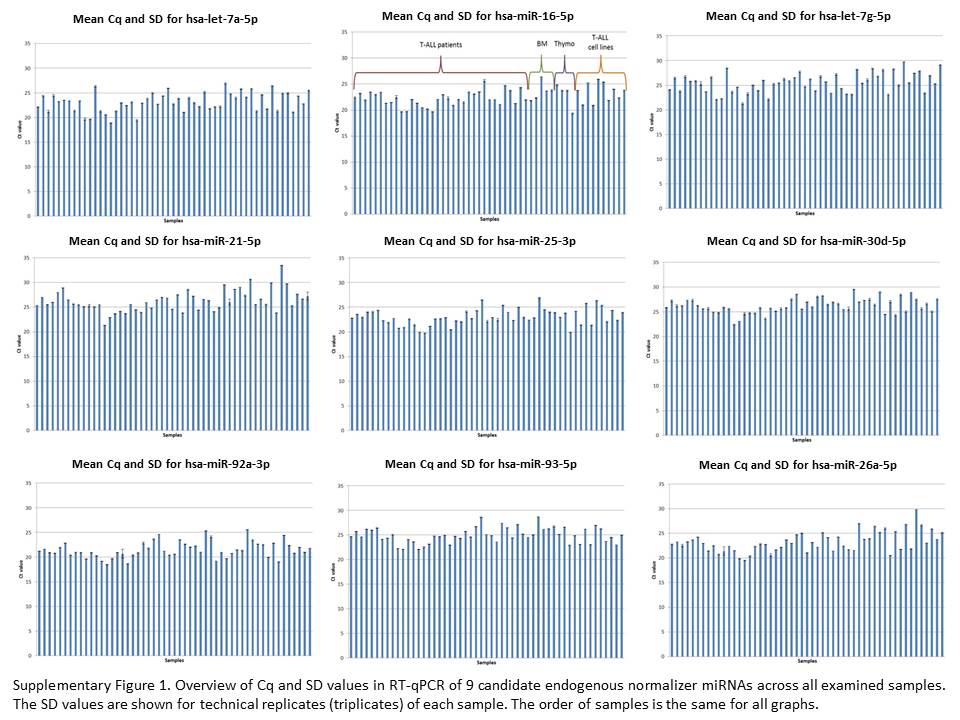


**Figure S1.** Overview of Cq and SD values in RT-qPCR for candidate endogenous normalizer miRNAs across all samples. The SD values are shown for technical replicates of each sample. The order of samples is the same for all graphs.


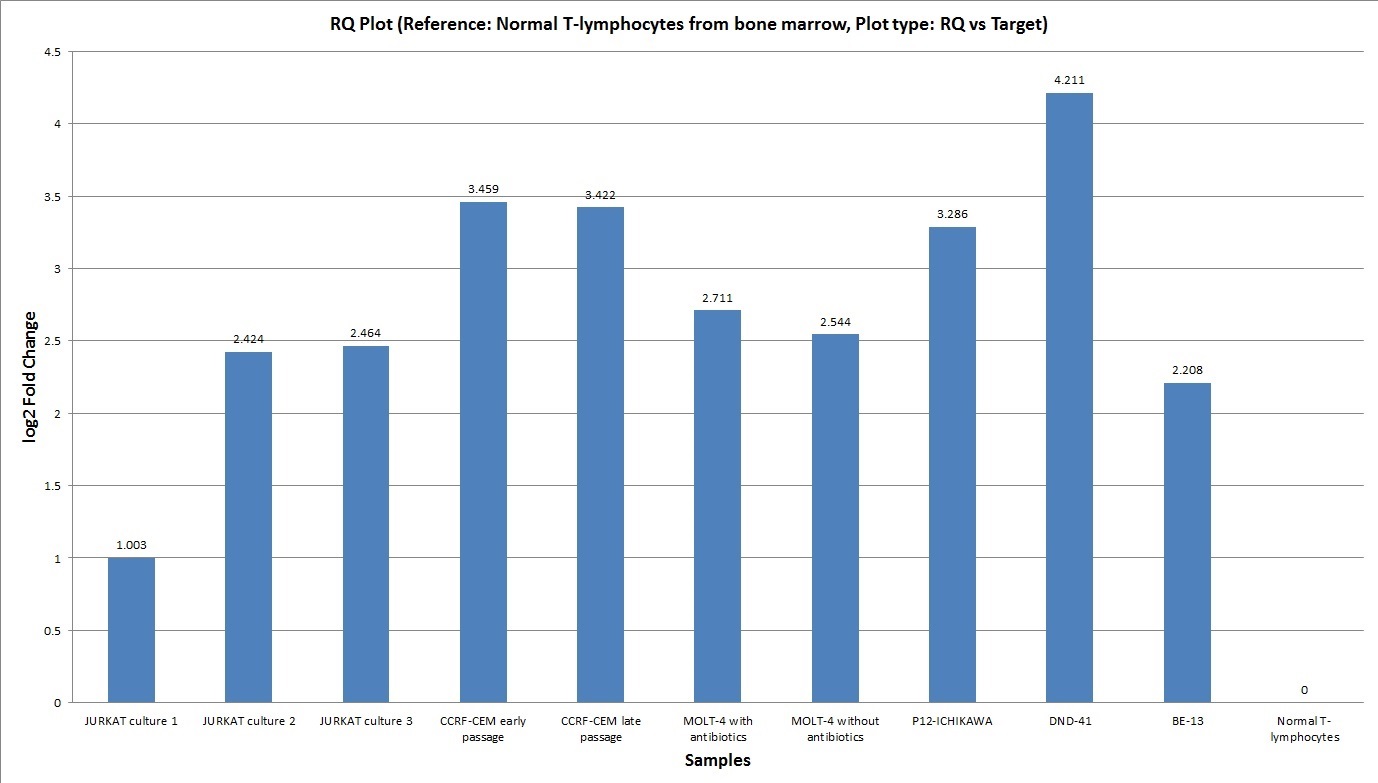


**Figure S2.** Relative expression of hsa-miR-128-3p in six T-ALL cell lines, cultured in various conditions.
The log2 fold change was calculated in reference to normal bone marrow T-lymphocytes.
