# Supplementary material for: Identification of Endogenous Control miRNAs for RT-qPCR in T-Cell Acute Lymphoblastic Leukemia
Source: Int J Mol Sci. 2018 Sep 20;19(10):2858. doi: 10.3390/ijms19102858 (PMC6212946; doi:10.3390/ijms19102858)
Supplement: Supplementary file 1 [file ijms-19-02858-s001.zip › 2018-08-06 Supplementary Tables.docx]

Identification of endogenous control miRNAs
for RT-qPCR in T-cell acute lymphoblastic leukemia

Monika Drobna ^1^, Bronisława Szarzyńska-Zawadzka ^1^, Patrycja Daca-Roszak ^1^, Maria Kosmalska ^1^, Roman Jaksik ^2^, Michał Witt ^1^, Małgorzata Dawidowska ^1^*

SUPPLEMENTARY TABLES

**Table S1.** Set of most stable miRNAs identified by iterative algorithm of stability based on miRNA-seq data

| **miRNA ID** | **Stability (NormFinder)** | **Mean Read Count** | **Log2 Fold Change** | **p-value** |
| --- | --- | --- | --- | --- |
| hsa-miR-1301-3p | 0.28 | 1315 | 0.040243367 | 0.980025402 |
| hsa-miR-185-5p | 0.27 | 2031 | 0.048790769 | 0.960760805 |
| hsa-miR-30d-5p | 0.25 | 98,732 | 0.055803957 | 0.937301436 |

**Table S2.** Raw Cq and SD values for candidate endogenous normalizer miRNAs across different culture types/conditions of T-ALL cell lines

| **T-ALL cell lines and culture type** | **Candidate EN miRNAs** | | | | | | | | | | | | | | | | | | | |
| --- | --- | --- | --- | --- | --- | --- | --- | --- | --- | --- | --- | --- | --- | --- | --- | --- | --- | --- | --- | --- |
|  | **let-7a-5p** | | **let-7f-5p** | | **let-7g-5p** | | **miR-16-5p** | | **miR-21-5p** | | **miR-25-3p** | | **miR-30d-5p** | | **miR-92a-3p** | | **miR-93-5p** | | **miR-26a-5p** | |
|  | **Cq** | **SD** | **Cq** | **SD** | **Cq** | **SD** | **Cq** | **SD** | **Cq** | **SD** | **Cq** | **SD** | **Cq** | **SD** | **Cq** | **SD** | **Cq** | **SD** | **Cq** | **SD** |
| **JURKAT culture 1** | 24.9 | 0.14 | 25.9 | 0.14 | 27.4 | 0.14 | 25.9 | 0.14 | 33.5 | 0.14 | 26.3 | 0.14 | 28.9 | 0.14 | 24.4 | 0.14 | 26.9 | 0.14 | 29.8 | 0.14 |
| **JURKAT culture 2** | 24.9 | 0.11 | 26.3 | 0.11 | 27.8 | 0.11 | 25.4 | 0.11 | 29.7 | 0.11 | 25.3 | 0.11 | 27.5 | 0.11 | 22.3 | 0.11 | 26.2 | 0.11 | 29.8 | 0.11 |
| **JURKAT culture 3** | 21.1 | 0.07 | 21.7 | 0.07 | 23.3 | 0.07 | 21.8 | 0.07 | 25.2 | 0.07 | 22.0 | 0.07 | 25.6 | 0.07 | 20.8 | 0.07 | 23.7 | 0.07 | 29.8 | 0.07 |
| **MOLT-4 with antibiotic** | 24.3 | 0.07 | 25.9 | 0.07 | 26.9 | 0.07 | 24.0 | 0.07 | 27.6 | 0.07 | 24.3 | 0.07 | 26.6 | 0.07 | 22.0 | 0.07 | 24.5 | 0.07 | 29.8 | 0.07 |
| **MOLT-4 without antibiotic** | 22.7 | 0.09 | 24.3 | 0.09 | 25.3 | 0.09 | 22.3 | 0.09 | 26.6 | 0.09 | 22.3 | 0.09 | 25.0 | 0.09 | 21.0 | 0.09 | 23.0 | 0.09 | 29.8 | 0.09 |
| **CCRF-CEM early passage** | 26.4 | 0.20 | 28.7 | 0.20 | 29.8 | 0.20 | 25.2 | 0.20 | 29.9 | 0.20 | 25.8 | 0.20 | 28.4 | 0.20 | 22.8 | 0.20 | 26.2 | 0.20 | 29.8 | 0.20 |
| **CCRF-CEM late passage** | 21.7 | 0.07 | 23.7 | 0.07 | 25.0 | 0.07 | 21.0 | 0.07 | 25.5 | 0.07 | 21.4 | 0.07 | 24.3 | 0.07 | 19.9 | 0.07 | 23.1 | 0.07 | 29.8 | 0.07 |
| **P12-ICHIKAWA** | 25.5 | 0.14 | 27.5 | 0.14 | 29.1 | 0.14 | 23.8 | 0.14 | 27.2 | 0.14 | 23.9 | 0.14 | 27.5 | 0.14 | 21.8 | 0.14 | 24.9 | 0.14 | 29.8 | 0.14 |
| **DND-41** | 21.3 | 0.13 | 23.3 | 0.13 | 25.5 | 0.13 | 20.9 | 0.13 | 23.8 | 0.13 | 21.3 | 0.13 | 25.0 | 0.13 | 19.0 | 0.13 | 23.0 | 0.13 | 29.8 | 0.13 |
| **BE-13** | 24.6 | 0.04 | 27.0 | 0.04 | 28.2 | 0.04 | 23.7 | 0.04 | 26.6 | 0.04 | 24.2 | 0.04 | 27.1 | 0.04 | 22.5 | 0.04 | 24.9 | 0.04 | 29.8 | 0.04 |

Cq and SD values represent mean values for technical replicates; culture 1, 2, and 3 of JURKAT represent three independent cultures of the same cell line

**Table S3.** Individual rank of most stably expressed candidate ENs for Comparative Delta Ct stability algorithm

| **miRNA name** | **Delta Ct ranking stability score** |
| --- | --- |
| hsa-miR-16-5p | 2.11 |
| hsa-miR-30d-5p | 2.71 |
| hsa-miR-25-3p | 2.99 |
| hsa-let-7g-5p | 3.98 |
| hsa-let-7a-5p | 4.36 |
| hsa-miR-93-5p | 4.53 |
| hsa-let-7f-5p | 4.58 |
| hsa-miR-92a-3p | 5.66 |
| hsa-miR-21-5p | 8.74 |
| hsa-miR26a-5p | 10 |

The stability score value is inversely proportional to the stability of gene expression. Ct values are equivalent to Cq values.

**Table S4.** Individual rank of most stably expressed candidate ENS for BestKeeper stability algorithm

| **miRNA name** | **BestKeeper ranking stability score** |
| --- | --- |
| hsa-miR-30d-5p | 1.22 |
| hsa-miR-92a-3p | 1.31 |
| hsa-miR-93-5p | 1.31 |
| hsa-miR-25-3p | 1.32 |
| hsa-miR-16-5p | 1.35 |
| hsa-let-7a-5p | 1.52 |
| hsa-let-7g-5p | 1.55 |
| hsa-miR-21-5p | 1.62 |
| hsa-let-7f-5p | 1.66 |
| hsa-miR26a-5p | 1.97 |

The stability score value is inversely proportional to the stability of gene expression.

**Table S5.** Individual rank of most stably expressed candidate ENS for NormFinder stability algorithm

| **miRNA name** | **NormFinder ranking stability score** |
| --- | --- |
| hsa-miR-16-5p | 0.502 |
| hsa-miR-25-3p | 0.54 |
| hsa-miR-30d-5p | 0.719 |
| hsa-miR-93-5p | 0.791 |
| hsa-let-7a-5p | 0.834 |
| hsa-let-7g-5p | 0.942 |
| hsa-let-7f-5p | 0.967 |
| hsa-miR-92a-3p | 1.173 |
| hsa-miR-21-5p | 1.389 |
| hsa-miR26a-5p | 2.943 |

The stability score value is inversely proportional to the stability of gene expression.

**Table S6.** Individual rank of most stably expressed candidate ENS GeNorm stability algorithm

| **miRNA name** | **GeNorm ranking stability score** |
| --- | --- |
| hsa-let-7f-5p \| hsa-let-7g-5p | 0.494 |
| hsa-let-7a-5p | 0.679 |
| hsa-miR-16-5p | 0.914 |
| hsa-miR-25-3p | 1.002 |
| hsa-miR-30d-5p | 1.06 |
| hsa-miR-93-5p | 1.089 |
| hsa-miR-92a-3p | 1.141 |
| hsa-miR-21-5p | 1.25 |
| hsa-miR26a-5p | 1.615 |

The stability score value is inversely proportional to the stability of gene expression.

**Table S7.** The result of similarity analysis for hsa-miR-181a-5p.

|  |  |  |  | **Expression in miRNA-seq** | | |
| --- | --- | --- | --- | --- | --- | --- |
| **miRNA 1 name** | **miRNA 2 name** | **Mature sequence similarity Aln score** | **Seed sequence identity** | **Pearson’s correlation** | **miRNA 1 mean read count** | **miRNA 2 mean read count** |
| hsa-miR-181a-5p | hsa-miR-181b-5p | 21.94 | TRUE | 0.77 | 155,917 | 49,664 |
| hsa-miR-181a-5p | hsa-miR-181c-5p | 29.60 | TRUE | 0.57 | 155,917 | 128 |
| hsa-miR-181a-5p | hsa-miR-181d-5p | 14.06 | TRUE | 0.52 | 155,917 | 4436 |

The Aln score is proportional to similarity of mature miRNA sequences
