# Supplementary figures and images for: Identification of Endogenous Control miRNAs for RT-qPCR in T-Cell Acute Lymphoblastic Leukemia
Source: Int J Mol Sci. 2018 Sep 20;19(10):2858. doi: 10.3390/ijms19102858 (PMC6212946; doi:10.3390/ijms19102858)

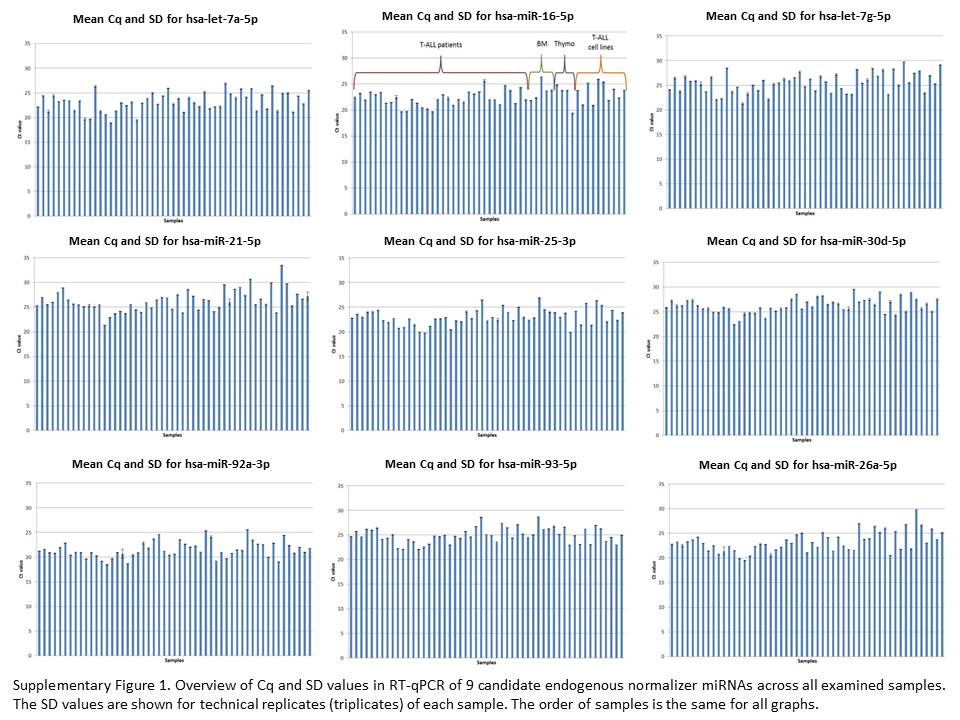

Supplement: Supplementary file 1 [file ijms-19-02858-s001.zip › Drobna et al. Suppl_ Figure 1_rev.jpg]

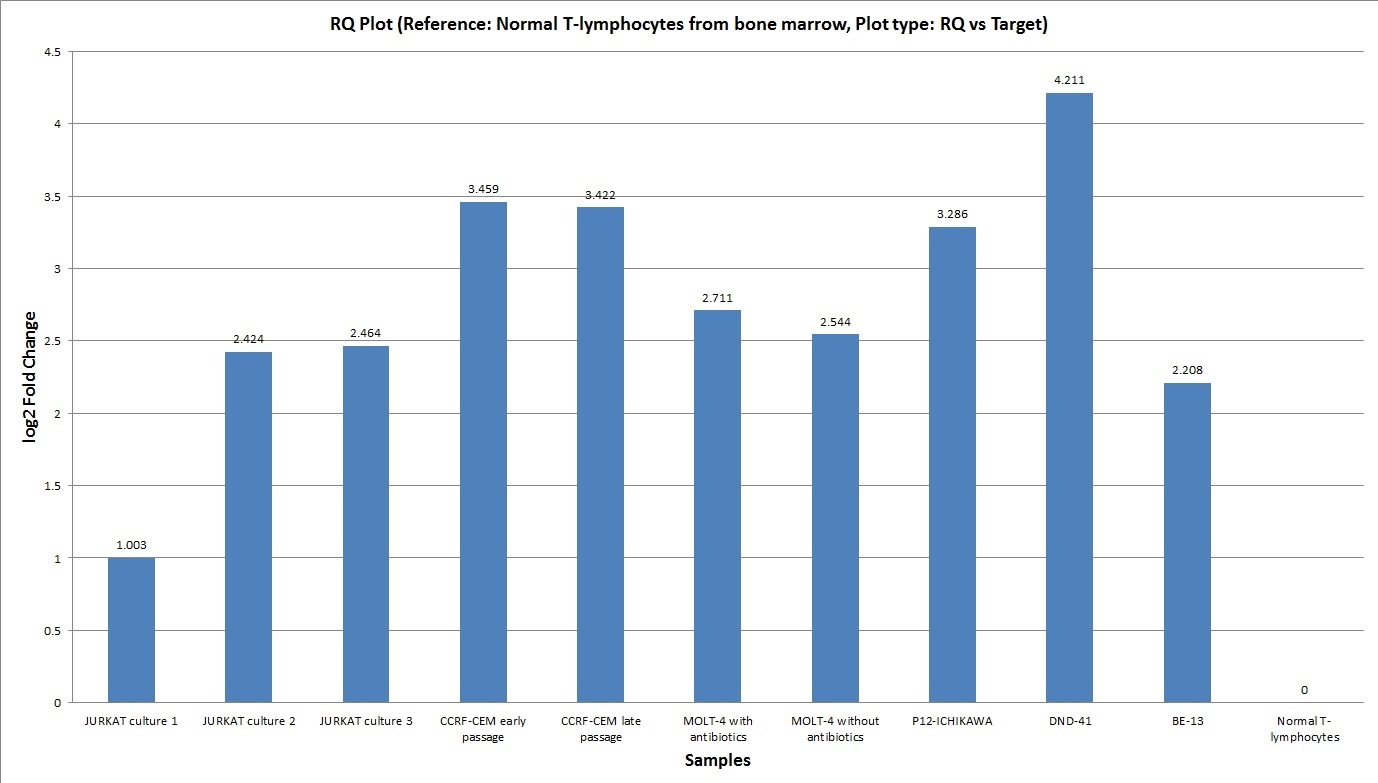

Supplement: Supplementary file 1 [file ijms-19-02858-s001.zip › Drobna et al. Suppl_Figure 2.jpg]
